# Using participatory action research methods to address epistemic injustice within mental health research and the mental health system

**DOI:** 10.3389/fpubh.2023.1075363

**Published:** 2023-03-21

**Authors:** Roisin Mooney, Clair Dempsey, Brian J. Brown, Frank Keating, Doreen Joseph, Kamaldeep Bhui

**Affiliations:** ^1^CHiMES Collaborative Group and World Psychiatry Associate Collaborating Centre, Department of Psychiatry, University of Oxford, Oxford, United Kingdom; ^2^School of Applied Social Sciences, De Montford University, Leicester, United Kingdom; ^3^Department of Law and Criminology, Royal Holloway University, London, United Kingdom; ^4^Nuffield Department of Primary Care Health Sciences, Wadham College, University of Oxford, Oxford, United Kingdom; ^5^Queen Mary University London Global Policy Institute, London, United Kingdom

**Keywords:** epistemic injustice, participatory research, psychiatric care, mental health, mental health act

## Abstract

In this paper, we describe a model of research practise that addresses epistemic injustice as a central objective, by valuing lived experience and addressing structural disadvantages. We set out here the processes we undertook, and the experiences of those involved in an attempt to transform research practise within a study known as Co-pact. We do not discuss the findings of the research. Rather, we wish to build expertise on how to address epistemic injustice and offer examples of participatory research processes, central values, and practical procedures that we implemented.

## 1. Defining epistemic injustice

The term ‘social epistemology’ has been used to describe attempts to understand the relationship between forms of knowledge and social life (1, 2). This includes the study of relationships between communities and knowledge, including the way groups might be organised so that they can create and deploy knowledge most persuasively and effectively (3). Fuller (4) maintains that knowledge can be seen as more than merely information about an independent phenomenon such as a risk, an illness or a treatment. The authors have investigated how forms of knowledge held by powerful groups intersect in intriguing and politically revealing ways with that of less powerful constituencies. Allied to this, Fricker (5) introduced the term ‘epistemic injustice’ to refer to a situation where someone (their perspectives and knowledge) is given less credence as a result of their social position. It can include actions such as excluding someone from participating in a discussion, silencing, or misrepresenting their views. In mental health care, for example, someone might be discredited, or their perspectives (lived experiences) might be regarded as unreliable. They may not have access to the same resources to make their views known and may be facing multiple disadvantages that require prioritisation for their immediate survival. When sharing knowledge, their perspective may not lead to any immediate benefit to the precarious situation in which they live and therefore may not be a priority.

Epistemic justice is about allowing or enabling people to think about their own experiences in ways that value those experiences, and support theorising about lived experience. The adoption of an epistemic justice framework recognises that knowledge is socially constructed and is valued irrespective of the source or social status of the person sharing their knowledge. The approach also endorses the view that multiple perspectives might exist and appear contradictory, but might all be valid and together to represent a more complete picture. This is an important acknowledgement when conducting qualitative research with heterogeneous populations, especially when diverse identities and contexts introduce contrasts in what matters and what is at stake.

As a corollary of this, Vaditya (6) writes of ‘epistemic domination’ which exists ‘not because of objectivity or universality’ of expert-led knowledge, but because its originators hold a ‘privileged location within a historical, material and social setup of dominant power relations’ (6, p. 272); in contrast, a ‘situated knowledge’ building process may help to end the epistemic oppression of disempowered groups. A similar notion of hermeneutical injustice is used to describe what happens when some groups are epistemically marginalised and excluded from being fully involved in the interpretation of knowledge that informs programme design and policy development (7, p. 163). This is not the same as prejudice or deliberate discrimination on the part of the actors in health and social care. Instead, the injustice results from ‘socioepistemic structures’ (8) which collectively disadvantage members of certain groups such that their opinion appears less credible or intelligible than that of others. More commonly, they are not central to knowledge production and interpretation. Marginalisation of this nature may lead to misunderstanding, imperfect and inaccurate ideologies, ‘epistemic oppression’ (9), and perhaps a lack in the progression of how information is used and understood; ‘hermeneutical death’ (10).

Fricker (5) has described two types of epistemic injustice, both based on wrongs done to someone in their capacity as a knower. The first type is testimonial injustice, which occurs when prejudice causes the hearer to assign a lower level of credibility to a speaker’s word (5). The second type, hermeneutical injustice, happens when there is a gap in collective interpretative resources that leads to someone being disadvantaged when making sense of their social situation (5). Whilst testimonial injustice refers to the lack of credibility placed on a person by others, hermeneutical injustice refers to more structural prejudice, for example within a culture or organisation. A health assessor who does not recognise a person’s identity, history, and forms of lived adversity (racism, poverty and unemployment), and thus diminishes the importance of these, demonstrates testimonial injustice. However, policies or guidance that prohibit asking about identity and racism, stipulates time limited assessments, or removes access to interpreters, are examples of hermeneutical injustice. Both are common in the mental health experiences and outcomes of black and minority ethnic groups in the United Kingdom, Europe, and North America (11).

## 2. Epistemic injustice in health care

Testimonial injustice occurs within the health system when healthcare professionals (including those in mental health care) are assigned more credibility than those living with a condition. Obviously, this is not inevitable, and depends on who is listening and their sensitivities. In clinical practise, research and mental health tribunals, professionals intend to hear the patient’s view; however, their automatic thought processes and experiences may drive them to subtly negate or devalue the perspectives of the patient. The lived experience of a mental health patient can be dismissed as subjective due to experiential nature of their symptoms. Furthermore, the presence of hallucinations and delusions can be used to devalue knowledge beyond the symptoms that the patient has to share. Even though the content may hold meaning, this may be dismissed as a pathology. In contrast, the healthcare professional may be judged as having objectivity, expertise, authority, and professional credibility. An example of hermeneutical injustice within the healthcare system and in research is apparent when some patient populations are described as ‘hard to reach’, justifying their exclusion. The healthcare system as a structure, with policies and procedures, struggles to meet the needs of these populations, with responsibility placed for the lack of engagement on the patient. This may not be overt prejudice but rather exclusionary in nature. The patients themselves may not be aware of the structural inequalities. Hacking describes that the way we see things in the world become facts, and that we behave as if these facts are real, even though they are really born of a specific niche in time, political and social context (12). Some may see distress or a health condition as an entirely embodied biological phenomena rather than understanding these concepts as a product of history, the environment, and past and contemporary adversity, or taking an eco-social and development lens.

## 3. Co-pact case study

The Co-pact study recruited participants from racialised communities, who had been detained under the Mental Health Act (1983) in the last 2 years (13). The study aims to change local and national policy informing the current reform of the Mental Health Act. The protocol for how this work is being conducted has been published (13). Here we provide some rationale and pragmatic examples as to the choices made in implementing our protocol that speak to acknowledging and reducing epistemic injustice in mental health research.

We were interested in participants’ experiences of being detained under the powers of the Act, as a starting point. Importantly, we consider these ‘experience data’ as important forms of valid and authentic knowledge that represent and reveal a real world occupied by the participant. Listening to such perspectives may help further the understanding of how to prevent detention in the future, and which structural and interpersonal processes lead to detention. We anticipate novel processes and mechanisms will be revealed, as marginalised world views rarely enter homogenised and normalised accounts of research data. These experiences might be dismissed as subjective because the research takes a qualitative form in which sampling is often purposive and not generalisable. Hierarchies of evidence may be invoked to diminish or discredit these views, privileging more normative and conventional research, in which marginalised groups are under-represented. These are all points or arguments that might be invoked to justify epistemic injustice. Thus, promoting experience data as a legitimate source of knowledge to inform both practise, service design, and policy was our first step.

The question of whose knowledge is given credence, and therefore worth and acceptability, needs to recognise that someone’s personal experience, their truth, cannot be denied and that it has legitimate value. Co-pact employed photovoice methodology (14) to provide an opportunity to counter such thinking that is prevalent in mental health research which informs mental health systems. Instead, our study demonstrated the value of the information and experiences that patients contribute to the conversation. This experiential knowledge is valid in and of itself because it aids recovery, wellbeing and enhances self-esteem, as well as reinforces the importance of being valued as a human being. Having recognition is validation of self-worth (15). Such knowledge is crucial for training mental health staff and can enhance decision-making. From a social epistemological perspective patient voice becomes acceptable, worth listening to, and acting upon. It is serious knowledge.

Whilst there is much discussion about how participatory action research is defined (16), and what is considered as a participatory process, we consider that this work fits with the participatory action research paradigm. They way in which photovoice workshops were conducted, enacted participatory process, and built capacity amongst participants. The outputs from the photovoice workshops will inform a series of co-design workshops, resulting in action both at a local, or community level and at a national level. In person exhibitions to further engage policymakers will be co-produced with participants.

Eliciting authentic experiential data is not straightforward. We might anticipate people would be distressed if asked to share information verbally or may wish to avoid reminders (as with all traumatic events). We employed creative methods, specifically photo-elicitation around which a person might progressively narrate and construct a story of their experiences (photovoice) which are not immediately available for sharing, or when sharing is attempted, are overwhelming which leads to defensive avoidance. Creative methods are emotionally and behaviourally activating, allow for perspective taking and engage different brain regions. These processes enable traumatic or adverse memories to be activated, held non-verbally and worked through, as part of the narration process. As demonstrated in previous photovoice studies, the approach might be helpful and empowering to participants (17), if sharing their experience and deepening reflection facilitates improved self-understanding through taking a different perspective (18, 19), particularly for racialised populations (20).

It was important within the internal structure of the team that there was not a dominant narrative that steered the conversation and that a diverse range of views were represented and heard in all conversations. Many discussions were held surrounding how we address power dynamics in different spaces, not only between researchers and participants or people with lived experience, but also in terms of ensuring more junior members of the team had a voice. With regards to race, and profession it was important that we recruited a diverse range of local Principal Investigators (PIs) in the eight NHS trusts we were working with. We reached out to various networks in each trust to ensure that overall, we had men and women, different ethnicities and both psychologist and psychiatrists as PIs. This meant that in our team meetings a diverse range of perspectives were shared and heard. It is important for the sustainability of this type of work to build capacity, share knowledge across different NHS trusts and highlight best practise of how we can change the system to raise awareness of epistemic injustice.

People with experience of being detained in the last 2 years consented to participate in the three photovoice workshops. NHS ethics was obtained to conduct this research in eightNHS trusts in England. The first workshop was an introduction to the study (which incorporated training around the ethics of photography and the use of images), the aim of the second workshop was for individual participants to reflect on their experience and add captions to photographs that they had taken, and the final workshop allowed participants to share their experiences with each other. In the first workshop, participants’ experience and resulting knowledge of the mental health act, and surrounding systems were acknowledged by the researchers. The researcher’s lack of knowledge of living in the current climate with mental illness and being detained was also explored. Researchers were careful to identify their roles as providing a platform for participants to share their stories and be heard. It was important to consider the power dynamics between the research team and the participants. Although we framed the participants as being equal to the research team, there will always be inherent experiences and processes that set the two apart, for example the research team being employed by an academic institution. A member of the research team with lived experience of being detained under the MHA attended workshops where possible. Ideally, all workshops would have been co-facilitated with people with lived experience. However, limited resources and time meant that that wasnot feasible.

In between the first two workshops participants took photographs in response to prompts provided adapted from existing photovoice techniques (21), the researchers organised the workshops and made sure film from disposable cameras were developed. Participants were solely responsible for assigning context to the images in the second workshop, then shared their images and experiences with each other in the third workshop. Unlike a traditional facilitated discussion or focus group, the images acted as a focus point for participants to share specific personal experiences and enabled further sharing of experiences amongst participants. The emphasis of the discussion was on the participants’ agenda as set by the photographs they had taken, as opposed to having a form of topic guide. More detail on this process can be found in the protocol for the study (13).

The Co-pact researchers were also interested in investigating and exploring any inequalities experienced by participants, which may have been tied to race. There were concerns that participants would be reluctant to disclose any inequalities that they had observed or experienced if they perceived the research team as being associated with their local NHS Trust or employed by the NHS. The research team, with guidance from the patient and public involvement research group (PPIRG), designed the photovoice workshops to be inclusive, safe spaces for participants. Participants were also asked to provide feedback about each workshop they attended, by answering short surveys containing questions written by the PPIRG. In the first workshop, a presentation was given by the research team, which acknowledged racial inequalities within the mental health system and how the research methods being used were different to traditional methods, and thus hoped for a sustained impact. It was explained that participation in the study was envisaged as the beginning of a collaboration between the participants and research team, should participants wish to remain engaged with the study after the workshops. Beyond the study, it remains important that participants have the option to have their names associated with their photographs during dissemination should they want to, rather than defaulting to sharing anonymised photographs which may be disempowering.

Throughout the photovoice data collection, participants appeared to perceive the photovoice workshops as safe spaces where they were empowered to contribute their experiences in an authentic and meaningful manner. Despite anticipations around re-traumatisation, many safeguarding issues concerned circumstances where participants made disclosures that were unknown to their clinical teams (such as eating disorders and being groomed). Participants also disclosed incidents where they had racially abused a member of staff, or other patients on a psychiatric ward. This indicates that participants were in a space where they did not feel judged and were able to share their experiences as a legitimate form of knowledge that would be heard and taken seriously. Researchers were trained in photovoice techniques and had weekly sessions with the wider study team to reflect and debrief of the experiences of conducting the workshops.

## 4. Participatory research as a vehicle to address to epistemic injustice

Participatory action research (PAR) is primarily concerned with the democratisation of knowledge curation, by ensuring that community members participate throughout the research process to produce authentic outputs which ultimately influence change. Therefore, the principles of how knowledge is curated and shared align with those of an epistemic justice framework. First developed to engage communities in expressing lived experience to inform policy in the early 1990s (22). Photovoice is well recognised as a form of participatory action research that elicits both visual data (photographs) and narrative data (participants’ voices). Creative methodologies such as photovoice are useful to engage marginalised groups who have historically been subject to epistemic injustice, to encourage them to reframe and consider their experiences as legitimate form of knowledge that has the potential to inform policy and service design (20).

## 5. Participatory research and patient and public involvement

From inception, deliberate choices were made to exclude testimonial injustice and promote the voices of lived experience as an integral source of knowledge by using participatory action research methods of photovoice and co-design. The work conducted by Co-pact is not limited to the participants when providing a platform for voices from those with lived experience around detention (23). We have included carers in our PPIRG and advisory group, in which a vast number of professions are represented. The next phase of this work will entail local and national co-design events, in which we will engage several people from relevant communities. The approach taken here enables us to go beyond token service user consultation and fully enfranchise people with lived experience as knowledge creators.

Working within an epistemic justice framework alongside and promoting open interdisciplinary necessitated regular communication across the research team. Weekly meetings have been held throughout the programme of research to enable research team to learn and acknowledge complexity (24), sharing and coping with feelings of distress and discussing power dynamics, all of which can easily contaminate everyday team functions, particularly when focussed on delivering a funded research project. Researchers seeking to create environments where diverse contributions are acknowledged as legitimate forms of knowledge need to be prepared and take reflective supervision to ensure they sustain their health and wellbeing as well as that of the participants.

The study offset risk of testimonial injustice by ensuring people with lived experience of detention were included in the infrastructure of the study, at all levels. For example, the PPIRG is chaired by two Black women; one had experience of being a carer for someone with severe mental illness and one with experience of being detained under the Mental Health Act who was employed by the University of Oxford as a co-Investigator on the study. The Co-pact study also has an advisory board, co-chaired by a Black man, who is an academic with lived experience of detention and advocates for better mental health systems. They bring knowledge, experience, and expertise.

Members of the PPIRG, participants and the research team had joint ventures in raising the profile of Co-pact and our aims. They were interviewed on a radio show and have contributed to a video as a case study for exemplars of participatory work. One participant fervently vocalised how grateful to Co-pact he was for the opportunity to share his experience of detention, and by this hoped to influence service transformation. ‘Knowledge is power’ it is said by Francis Bacon, and knowledge from those who have been detained under the MHA, empowers them to influence positive change and radical transformation in research, policy and beyond—to society.

## 6. Conclusion

Approaching mental health research from a perspective grounded in social epistemology can have important benefits. It is commonplace that different actors in mental health care have different experiences and perspectives, and that these exist in a hierarchy of credibility. Typically, doctors, researchers, and allied professionals at the top, and informal carers and service users—especially those detained compulsorily under mental health legislation—are at the bottom. In the past, the knowledge of lay people has often been framed in terms which privilege professional understanding—notions such as ‘health beliefs’ or ‘mental health literacy’ are often explicitly formulated so as to bring laypeople into alignment with professional thinking. By contrast, taking participants’ accounts seriously as knowledge in their own right can enable us to appreciate it in its full complexity and yield genuinely emancipatory and humane opportunities in research and service development.

Participatory action research, and creative methodology such a photovoice may offer practical way to engage with and elevate the voices of people with lived experience in mental health research. In addition, embedding people with lived experience in the infrastructure of any research and facilitating continued and open dialogue around the interpretation and sharing of any findings in integral to addressing epistemic injustice.

## Data availability statement

The original contributions presented in the study are included in the article/supplementary material, further inquiries can be directed to the corresponding author.

## Author contributions

RM finalised the manuscript and integrated comments over several iterations. CD drafted the first version. RM, BB, FK, DJ, and KB contributed text, introduced edits to improve the final paper, and reviewed and agreed to the manuscript prior to submission. All authors contributed to the article and approved the submitted version.

## Funding

The Co-Pact study/project is funded by the National Institute for Health Research (NIHR) Policy Research Programme (NIHR201704). The views expressed are those of the authors and not necessarily those of the NIHR or the Department of Health and Social Care. KB and RM are Co-PIs, DJ is a Co-I, CD was employed on the project, and BB and FK sit on the project’s advisory board.

## Conflict of interest

The authors declare that the research was conducted in the absence of any commercial or financial relationships that could be construed as a potential conflict of interest.

## Publisher’s note

All claims expressed in this article are solely those of the authors and do not necessarily represent those of their affiliated organizations, or those of the publisher, the editors and the reviewers. Any product that may be evaluated in this article, or claim that may be made by its manufacturer, is not guaranteed or endorsed by the publisher.
